# Do the honeybee pathogens *Nosema ceranae* and deformed wing virus act synergistically?

**DOI:** 10.1111/1758-2229.12052

**Published:** 2013-04-10

**Authors:** Stephen J Martin, Jennifer Hardy, Ethel Villalobos, Raquel Martín-Hernández, Scott Nikaido, Mariano Higes

**Affiliations:** 1 School of Environment and Life Sciences, University of SalfordGreater Manchester, M5 4WT, UK; 2 Department of Molecular Biology and Biotechnology, University of SheffieldSheffield, S10 2TN, UK; 3 Department of Plant and Environmental Protection Sciences, University of Hawaii at ManoaHonolulu, HI, USA; 4 Centro Apicola Regional, Consejeria de Agricultura, Junta de Comunidades de Castilla-La ManchaMarchamalo, 19180, Guadalajara, Spain; 5 Instituto de Recursos Humanos para la Ciencia y la Tecnología (INCRECYT), Fundación Parque Científico y Tecnológico de AlbaceteAlbacete, Spain

## Abstract

The honeybee pathogens *Nosema ceranae* and deformed wing virus (DWV) cause the collapse of honeybee colonies. Therefore, it is plausible that these two pathogens act synergistically to increase colony losses, since *N. ceranae* causes damage to the mid-gut epithelial ventricular cells and actively suppresses the honeybees’ immune response, either of which could increase the virulence of viral pathogens within the bee. To test this hypothesis we exploited 322 Hawaiian honeybee colonies for which DWV prevalence and load is known. We determined via PCR that *N. ceranae* was present in 89–95% of these colonies, with no *Nosema apis* being detected. We found no significant difference in spore counts in colonies infected with DWV and those in which DWV was not detected, either on any of the four islands or across the entire honeybee population. Furthermore, no significant correlation between DWV loads (ΔC_T_ levels) and *N. ceranae* spore counts was found, so these two pathogens are not acting synergistically. Although the Hawaiian honeybees have the highest known prevalence of *N. ceranae* in the world, with average number of spores been 2.7 million per bee, no acute *Nosema* related problems i.e. large-scale colony deaths, have been reported by Hawaiian beekeepers.

## Introduction

It is well established that millions of honey bee colonies have been killed due to the global spread of the Varroa mite and its inter-action with deformed wing virus (DWV) (Martin *et al*., 2012). More recently honeybee colony losses have occurred across America and Europe that cannot be attributable to the Varroa mite, so unknown combinations of stressors are suspected (Potts *et al*., 2010). These could be various combinations of pathogens (Cox-Foster *et al*., 2007; Higes *et al*., 2009) or effects of pesticides and pathogens (vanEngelsdorp *et al*., 2009). For example, synergistic effects between various pesticides and the microsporidia fungal pathogen *Nosema ceranae* have been found, which in combination, were found to increase honey bee mortality in laboratory essays (Alaux *et al*., 2010; Vidau *et al*., 2011; Pettis *et al*., 2002). Although at a field scale any synergistic effect between DWV and *N. ceranae* was lacking (Hedtke *et al*., 2011). Studies looking at synergistic effects under natural field conditions between two or more pathogens are rare, since it is difficult to find situations where the prevalence and, more importantly, the load of a pathogens is known to exist at different levels within the same population. The accidental introduction of the Varroa mite to the Hawaiian Islands of Oahu and Big Island, but not Maui and Kauai has caused a large change in both the prevalence and load of DWV across only two of these islands (Martin *et al*., 2012). This provides a unique opportunity to investigate whether DWV and *Nosema* act synergistically to become more virulent when combined in the same colony. Both DWV (Schroeder and Martin, 2012) and *N. ceranae* (Higes *et al*., 2007; 2009) are known to kill honey bee colonies in their own right. Although *N. ceranae* has been implicated in the large-scale colony losses in Spain (Higes *et al*., 2009), its impact in other countries remains controversial (Higes *et al*., 2013). However, a synergistic effect between these two pathogens is highly plausible, since the obligate, spore-forming, intracellular parasite *N. ceranae* causes extensive damage to the mid-gut epithelial ventricular cells (Fries, 2010; Dussaubat *et al*., 2012). This could then allow viral pathogens, such as DWV, to pass more easily through the gut wall into the haemolymph which is supported by correlative evidence (Bromenshenk *et al*., 2010; Costa *et al*., 2011). The gut wall is an important barrier against viral pathogens, since oral transmission of viruses between bees are significantly less efficient than when a virus is injected directly into the haemolymph (Bailey and Ball, 1991). It has also been shown that *N. ceranae* can actively suppresses the immune response in honeybees (Chaimanee *et al*., 2012), which may also make *N. ceranae* infected colonies more susceptible to viral infections.

To test the hypothesis that *Nosema* and DWV act synergistically, we used our existing viral data from 322 colonies across the four main Hawaiian islands of Oahu (5 apiaries), Big Island (14 apiaries), Maui (4 apiaries) and Kauai (6 apiaries) (Martin *et al*., 2012). From these same colonies we surveyed for the prevalence of *Nosema apis* and *N. ceranae* via PCR and estimated *Nosema* load (spore count) in a subset of colonies in which DWV was either detected (DWV+) or not (DWV−).

## Results and discussion

Determination of the species of *Nosema* by PCR followed the methodology described by Higes and colleagues (2008) and Martín-Hernández and colleagues (2012) using a pool of 30 bees collected from the brood area from each colony at the same time the samples for DWV analysis were collected. Across all four major Hawaiian Islands *N. ceranae* was the only *Nosema* species detected in the 322 colonies surveyed. Of these 283 colonies (88%) and all but one of the 31 apiaries tested positive for *N. ceranae*. In five of the eight *N. ceranae* free colonies as determined by PCR, spores were detected under the microscope. This discrepancy is due to the known high degree of variability between *Nosema* subsamples (Botías *et al*., 2012a). This increased the prevalence to 89–95% assuming that spores were absent in all the other PCR-negative samples, or were present in 63% (i.e. five-eighths) of the remaining PCR-negative samples. Based on the PCR results no significant difference in *N. ceranae* prevalence between the four islands (Kruskal–Wallis, d.f. = 3, *H* = 7.64, *P* = 0.054) was found.

**Figure 1:**
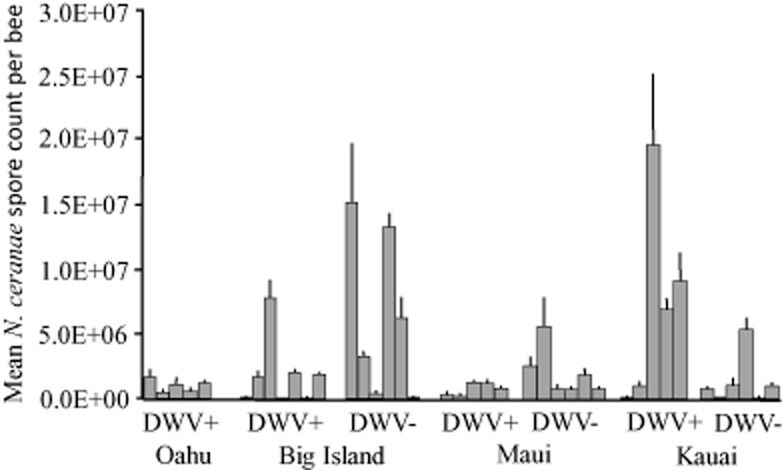
Shows the average *Nosema ceranae* counts per bee for 40 colonies across the four islands. Each bar represents a colony and error bars indicate one standard deviation. No significant difference in spore counts between colonies in which DWV was detected (DWV+) or not (DWV−) was found on any of the three islands where both groups exists. The DWV titre (mean number of DWV copies per bee and ΔC_T_ value) on each island had been previously calculated as Kauai (10^3.7^, −14), Maui (10^4.2^, −17), Big Island (10^3.5^, −15), and on Oahu (10^10^, 7) (see Martin *et al*., 2012 for details).

**Figure 2:**
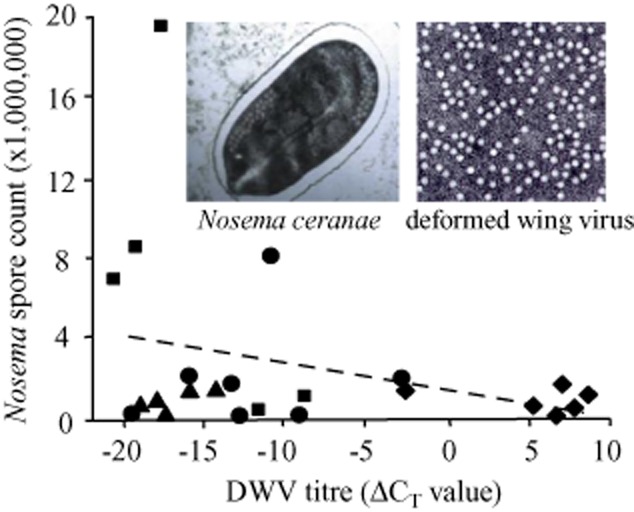
Shows the lack of a significant correlation between *Nosema ceranae* spore counts and DWV load, across the 23 colonies studied on Oahu (diamond), Maui (triangle), Big Island (circle) and Kauai (square). The higher the ΔC_T_ value the higher the amount of virus in that sample of bees, i.e. colony.

We selected two groups from each island from the 283 colonies that tested positive for *N. ceranae* in which DWV had been previously detected (DWV+) or not (DWV−). The long-term establishment of Varroa on Oahu meant that no DWV− colonies were present. The amount of DWV had been previously determined from a pooled sample of 30 bees and the ΔC_T_ values were used as an indication of viral load in each colony (for details see Martin *et al*., 2012). Our *N. ceranae* spore counts were based on pooled samples of 20 bees using the standard methods and were highly variable between colonies, as is typical for this species (Meana *et al*., 2010; Mulholland *et al*., 2012), but no significant difference (Kruskal–Wallis, d.f. = 3, *H* = 1.37, *P* = 0.7) was detected between the four islands. Across all samples the average and median spore counts were 2.7 million/bee and 1.1 million/bee respectively. We acknowledge that spores counts may not be the most accurate method to determine *N. ceranae* infection (Meana *et al*., 2010), but qPCR results that measure the actively reproducing vegetative state have been found to be significantly correlated with spore counts, although the level of correlation varied from strong (*r*^2^ = 0.74, Bourgeois *et al*., 2010) to weak (*r*^2^ = 0.26, Traver and Fell, 2011). Although, when high or moderate infection levels are present, 80% of the samples testing positive by qPCR also contained spores (Traver and Fell, 2011). No significant different in spore count between colonies infected with DWV or without DWV could be detected in any of the three islands populations (Mann–Whitney, Big Island *U* = 10, *P* = 0.14; Maui *U* = 22, *P* = 0.25; Kauai *U* = 7, *P* = 0.17) (Fig. 1) or when the data from all islands were combined (*U* = 150, *P* = 0.25, *n* = 41). Again no significant correlation (Spearman Rank, *n* = 24, *r* = −0.147, *P* = 0.5) between DWV viral load data (ΔC_T_ values) and *N. ceranae* spore counts was detected (Fig. 2). Therefore, while both DWV and *N. ceranae* are known to be lethal pathogens of honeybees when they occur at high loads (Higes *et al*., 2009; Martin *et al*., 2012), we found no significant effect, positive or negative, indicating that there was no synergistic or additive effect between DWV and *N. ceranae* in the Hawaiian honey bee population. Increased DWV prevalence and load associated with the spread of the Varroa mite corresponded with an increase in colony losses on Oahu and Big Island (Martin *et al*., 2012). However, the present data show that prevalence and load of *N. ceranae* were not significantly different from other islands (Maui and Kawai) where DWV prevalence and load remained low and no increase in colony losses were seen. As DWV prevalence and load is closely correlated with the presence of the Varroa mite (Martin *et al*., 2012), as expected there was also no synergistic effect between the presence of Varroa and *N. ceranae*. A weak correlation was reported between Varroa and *Nosema* in Germany, but this finding was based only on presence or absence data (Hedtke *et al*., 2011).

As *Nosema* has never been seen as a major problem among Hawaiian beekeepers it was generally assumed that *Nosema* levels would be low, i.e. below the economic threshold of 1 million spores per bee (El-Shemy and Pickard, 1989). Furthermore, due to import restrictions of honeybee colonies into the islands since the 1930s (Eckert, 1950), which were rigorously enforced after the arrival of Varroa on the US mainland in 1987, it was assumed that *N. apis* would be the dominant species. So it was unexpected that we detected only *N. ceranae* in Hawaii. It was originally believed that *N. ceranae* had recently expanded from its natural host *Apis ceranae* to include *Apis mellifera* (Higes *et al*., 2006; Huang *et al*., 2008), since it appeared to be replacing *N. apis* in several populations across Europe (Klee *et al*., 2007). Although more recent European studies found no evidence that replacement is occurring (Paxton *et al*., 2007; Gisder *et al*., 2010; Martín-Hernández *et al*., 2012; Forsgren and Fries, 2013). In the USA there are confirmed infections of *N. ceranae* dating back to 1985 (Traver *et al*., 2012, Accession No. FJ416497.1) and it was already widespread in the mid-1990s (Chen *et al*., 2008). Therefore, the current situation on Hawaii is very similar to that on the US mainland, but the question of how and when *N. ceranae* first arrived in Hawaii remains open.

Hawaii currently has the highest *N. ceranae* prevalence of any known honeybee population with this study detecting it in 89–95% of the 322 study colonies and well above the average value of 57% for the US mainland (Rennich *et al*., 2012). In the warmer climates of Southern Europe *N. ceranae* and not *N. apis* dominates (Martín-Hernández *et al*., 2012), which would help explain the dominance of *N. ceranae* in Hawaii with its subtropical climate. However, *N. ceranae* was also the dominate species in regions with a cold climate such as Canada and Minnesota (Williams *et al*., 2008), Germany (Gisder *et al*., 2010), Scotland (Bollan *et al*., 2013) and the Balkans (Stevanovic *et al*., 2011). However, in the cold climate of Sweden and Norway *N. apis* was dominate (Forsgren and Fries, 2013), indicating that climate is a poor predictor of *Nosema* species.

The average spore count of 2.7 million per bee in Hawaii, which is typical for many studies, is well below the 10s-100s of millions detected from in-hive bees from CCD colonies (Cox-Foster *et al*., 2007) or 10s of millions in those that died in Spain (Higes *et al*., 2008). The spore counts presented in this study are expected to be underestimates, since they were conducted on bees collected from the brood combs (nurse or in-hive bees) that have lower counts than foraging bees (El-Shemy and Pickard, 1989; Higes *et al*., 2008; Smart and Sheppard, 2012), although Traver and colleagues (2012) found that there was no significant difference between in-hive and foragers in *N. ceranae* infection levels based on q-PCR results, which detects the earlier vegetative stage.

Despite the almost universal occurrence of *N. ceranae* across Hawaii combined with high loads, and potential increase in reproductive potential due to high climate temperatures (Martín-Hernández *et al*., 2009), nosemosis has never been considered a major problem in Hawaii and few beekeepers treat for it when questioned while collecting bee samples for this study. The lack of reported acute effects on Hawaii i.e. colony death is similar to other large-scale studies looking at the impact of *N. ceranae* on colony losses. For example, in Uruguay Invernizzi and colleagues (2009) found no correlation between the arrival of *N. ceranae* and either increasing microsporidia loads or increased colony losses. The studies of Traver and Fell (2011) and Traver and colleagues (2012) found no correlation between colony strength and infection level, despite 70% of their 300 study colonies being infected by *N. ceranae*. Likewise a German study of 220 colonies found no relationship between colony mortality and detectable levels of *N. ceranae* infection (Gisder *et al*., 2010) as did a Spanish study involving 77 apiaries (Fernández *et al*., 2012). It is only in Spain that *N. ceranae* has been directly associated with large-scale colony losses (Higes *et al*., 2009). It is currently unclear why these differences exist (Higes *et al*., 2013). It may be due to *N. ceranae* strain differences as found in DWV (Martin *et al*., 2012). Higes and colleagues (2013) observed that colony losses were not generally reported from studies conducted in colder areas; however, colony losses were also not reported from Hawaii with its warmer climate.

Although potentially there could be a synergistic effect between DWV and *N. ceranae* this idea has not been supported by this study, nor the larger ‘prevalence only’ study of Hedtke and colleagues (2011). Furthermore, the study of Costa and colleagues (2011). found no significant correlation between *N. ceranae* and DWV at the whole bee level. In the midgut they also found no evidence of synergistic effects, but possibly an antagonistic effect, since a negative correlation between *N. ceranae* and DWV was found. Dussaubat and colleagues (2012), also mentioned they found no synergistic effect but no details are given. Despite this study showing the Hawaiian honeybee population has the highest prevalence of *N. ceranae* ever found, why this situation exists is currently unclear. Climatic conditions in Hawaii allow honeybee brood to be produced continuously, so turnover rates of workers and queens are high and this may affect *N. ceranae* levels (Botías *et al*., 2012b). Furthermore, *N. ceranae* could be having an unseen subclinical effect, but further studies are required to discover the real impact, if any, of this pathogen in subtropical climates such as Hawaii.
